# Fortifying plant fortresses: siderophores in defense against *Cercospora* leaf spot disease in *Vigna radiata L*.

**DOI:** 10.3389/fmicb.2024.1492139

**Published:** 2025-01-27

**Authors:** Anushree Kamath, Abhishek Sharma, Arpit Shukla, Paritosh Parmar, Dhara Patel

**Affiliations:** ^1^Department of Biotechnology and Bioengineering, Institute of Advanced Research, Koba Institutional Area, Gandhinagar, India; ^2^Alimentary Pharmabiotic Centre, University College Cork, Cork, Ireland; ^3^Cancer Research Centre, University College Cork, Cork, Ireland

**Keywords:** siderophore, plant growth-promoting rhizobacteria, leaf spot disease, plant defense, induced systemic resistance

## Abstract

Siderophores, specialized iron-chelating molecules produced by *Bacillus amyloliquefaciens* D5, were investigated for their role in enhancing plant defense mechanisms against *Cercospora canescens* in mung bean (*Vigna radiata L.*). Siderophores were extracted and purified using Amberlite XAD-4 and applied to plants at concentrations of 5, 10, and 15 µg/mL, followed by pathogen inoculation. The treatments significantly influenced enzymatic activities and defense-related gene expression. On Day 6, peroxidase (POD) activity reached its highest value of 0.563 in the SP15 (siderophore + pathogen at 15 µg/mL) treatment, with S15 (siderophore-only at 15 µg/mL) showing a lower but significant increase of 0.453, while control groups remained unchanged. Polyphenol oxidase (PPO) activity peaked in SP15 (0.10 U/mL), followed by S15 (0.08 U/mL), highlighting the role of these treatments in enhancing stress responses. Chitinase activity was significantly elevated in SP15 on Day 6, with a sustained response through Day 8, while no significant change was observed in the control group. Total phenolic content was highest in SP15 (100 µg/mL), showing a a ramified immune response whereas S15 recorded 80 µg/mL, significantly above the control. Gene expression analysis further demonstrated the effectiveness of siderophore and siderophore + pathogen treatments. Catalase expression was upregulated by 21.1-fold in siderophore-only treatment and amplified to 25.9-fold in SP15. Epoxide hydrolase (EH) gene expression increased by 77.3-fold in S15 and further synergized to over 90-fold in SP15. Similarly, PR10 expression showed moderate upregulation in S15 and significantly higher levels in SP15, reflecting enhanced pathogen defense. Calmodulin (CAL) gene expression was moderately regulated in S15 but significantly amplified in SP15. These findings underscore the dual role of siderophores in nutrient acquisition and as potent elicitors of plant defenses, highlighting their potential as bio-stimulants. Field trials are essential to validate these results under natural conditions and optimize their use in agriculture.

## Introduction

In the quest for sustainable agricultural practices, researchers continually explore novel strategies to enhance plant growth and defense against pathogens. Among the myriad of approaches, harnessing the potential of plant growth-promoting bacteria (PGPB) has garnered significant attention due to their ability to enhance nutrient availability, suppress pathogens, and induce systemic resistance, making them a cornerstone of sustainable agricultural practices (Ha-Tran et al., 2021). One of the key mechanisms by which PGPR stimulate plant growth is through the solubilization of mineral nutrients, such as phosphorus and iron, making them more accessible to plants. PGPR produce siderophores, small molecules that chelate iron, making it available to plants in environments where it is limited (Goswami et al., 2016; Vocciante et al., 2022). PGPR also contribute to plant growth promotion through the production of phytohormones, such as auxins, cytokinins, and gibberellins, which regulate various aspects of plant growth and development. These hormones stimulate root elongation, enhance nutrient uptake, and promote the formation of lateral roots, leading to increased root surface area and improved nutrient acquisition efficiency (Santoyo et al., 2021; Jeyanthi and Kanimozhi, 2018; Prasad et al., 2019). Additionally, PGPR-mediated induction of systemic resistance pathways primes plants to defend against pathogen attacks, enhancing their resilience to biotic stresses. Furthermore, PGPR can suppress plant pathogens directly through mechanisms such as competition for nutrients and space, antibiosis, and the induction of systemic resistance in plants. By colonizing the rhizosphere and outcompeting deleterious microorganisms for resources, PGPR produce signaling molecules, including volatile organic compounds and quorum-sensing molecules as well as secondary metabolites which modulate plant gene expression and systemic defense responses. In response, plants secrete specific compounds, such as flavonoids and phenolic acids, that attract beneficial microorganisms to the rhizosphere and enhance their colonization. *Bacillus amyloliquefaciens*, a ubiquitous soil bacterium, has emerged as a promising candidate owing to its multifaceted beneficial effects on plant health. These include enhancing nutrient uptake, producing antimicrobial compounds, inducing systemic resistance pathways, and secreting siderophores that chelate iron, thus improving plant resilience against pathogens (Wu et al., 2024; Márquez et al., 2020).

As a prolific producer of bioactive compounds, this Gram-positive bacterium exerts beneficial effects on plant health through various mechanisms. *B. amyloliquefaciens* enhances plant defense mechanisms against phytopathogens through the production of antimicrobial compounds and the induction of systemic resistance pathways. Notably, the secretion of siderophores, small iron-chelating molecules, plays a pivotal role in the interaction between *B. amyloliquefaciens* and plants. In a study reported by Dimopoulou et al., 2021 the siderophore bacillibactin, produced by *Bacillus amyloliquefaciens* MBI600, under iron-limiting conditions, and its biocontrol activity against both bacterial and fungal pathogens was explored. The findings demonstrate that bacillibactin contributes to antimicrobial activity by inhibiting the growth of *Pseudomonas syringae pv. tomato* and enhancing antifungal activity. Additionally, the biosynthesis of bacillibactin is tightly regulated with genes related to microbial competition and fitness, further supporting its role in biocontrol. In comparison, the isolate *Bacillus amyloliquefaciens* D5, identified in our study, also produces significant amounts of siderophores, contributing to its biocontrol properties and potential of inducing systemic resistance in plants against pathogen invaders. Induced systemic resistance (ISR) is triggered by plant growth-promoting rhizobacteria (PGPR) and other microorganisms, primarily through jasmonate- or ethylene-sensitive pathways. This mechanism, which helps plants resist various pathogens, is activated by PGPR strains like *Pseudomonas*, *Bacillus*, and *Trichoderma*. ISR operates similarly to pathogen-induced defense mechanisms but involves complex microbial signals and determinants that remain poorly understood. Future research aims to explore the molecular regulation of ISR by addressing specific questions, such as which microbial signals most effectively trigger resistance and how these signals interact with plant hormonal pathways. Investigating the temporal dynamics of ISR activation and identifying genetic determinants in both plants and microbes that enhance resistance could guide the development of tailored biocontrol strategies (Pradhan et al., 2023; Luo et al., 2022).

Siderophores are essential for iron acquisition in environments where this vital micronutrient is limited. However, beyond their role in iron uptake, siderophores exhibit diverse functions in plant-microbe interactions, including plant growth promotion and defense mechanisms facilitates iron acquisition from the rhizosphere, thereby alleviating iron deficiency stress in plants (Ghazy and El-Nahrawy, 2021).

Iron, an essential micronutrient for various physiological processes, is often limited in alkaline and calcareous soils, adversely impacting plant growth and development. By solubilizing iron through chelation, siderophores enhance its availability for plant uptake, consequently ameliorating iron deficiency-induced chlorosis and promoting overall plant vigor. Furthermore, siderophores play a pivotal role in modulating the rhizosphere microbiome and enhancing plant resistance against phytopathogens. The competitive advantage conferred by siderophore-producing bacteria enables them to outcompete deleterious microbes for iron, thereby suppressing their proliferation and pathogenicity (Aznar and Dellagi, 2015). Moreover, siderophores exhibit direct antagonistic effects against phytopathogens by sequestering iron essential for their growth and virulence, thus impeding their pathogenicity. Beyond their role in iron acquisition and pathogen suppression, siderophores serve as signaling molecules mediating plant-microbe communication and eliciting systemic resistance responses in plants. Through intricate signaling pathways, siderophores prime plants to mount a more robust defense response upon subsequent pathogen encounters, thereby enhancing their resistance to biotic stresses. Moreover, siderophore-mediated induction of systemic resistance pathways stimulates the production of secondary metabolites and phytohormones associated with plant defense, further fortifying plants against pathogen invasion. This study aims to investigate the role of siderophores extracted from *Bacillus Bacillus amyloliquefaciens* D5 as elicitors of plant defense mechanisms and enhancers of resilience under biotic stress conditions. It focuses on evaluating their ability to activate defense enzymes, regulate gene expression, and influence protein expression associated with systemic resistance pathways in *Vigna radiata*. By elucidating these biochemical and molecular responses, the research highlights the multifaceted potential of *Bacillus amyloliquefaciens*-derived siderophores in suppressing phytopathogens through rhizosphere modulation, and inducing systemic resistance. These findings highlight the significance of siderophores as eco-friendly elicitors and bio-stimulants, offering sustainable and effective strategies for agricultural biotechnology.

## Materials and methods

### Bacterial and fungal strain

In this study, *Bacillus amyloliquefaciens* D5, previously isolated from a soil sample collected from the Vagadkhol area, was used. The strain was identified through 16S rRNA sequencing, and its taxonomic position was confirmed using a phylogenetic tree constructed with closely related sequences. The culture has been submitted to GenBank with the accession number OQ536061, providing a validated reference for its identity.

The fungal strain used was *Cercospora canescens* MTCC no. 10835. The culture was routinely maintained on Potato Dextrose Agar.

### Extraction and purification of siderophore

Siderophore production by *Bacillus amyloliquefaciens* D5 was carried out in an iron-depleted succinic acid medium containing potassium phosphate, magnesium sulfate, ammonium sulfate, and succinic acid. Cultures were inoculated at an initial density of 10^6^ CFU/mL and incubated at 28 ± 2°C with continuous agitation (120 rpm) for 24–48 h. Optimization of production conditions, including pH, temperature, and iron concentration, was performed prior to purification. Following incubation, the culture supernatant was harvested by centrifugation (5,000 rpm, 15 min), and siderophore content was quantified using the Chrome Azurol S (CAS) assay. The CAS-positive supernatant was concentrated using a rotary vacuum evaporator at 50°C (pH 6.0). Purification was performed using an Amberlite XAD-400 resin column prepared and activated as described by Sayyed et al. (2006) and Nithyapriya et al. (2021). The supernatant was loaded onto the column at a flow rate of 1–2 mL/min until column saturation, indicated by browning. The column was sequentially washed with distilled water, and siderophores were eluted using ethyl acetate as a solvent (Budzikiewicz, 1993). Filtrate, water wash, and eluted fractions were collected and screened for siderophore activity using the CAS assay.

### Siderophore induced biochemical enzyme activity

The biochemical enzyme activity induced by siderophores was evaluated using *Vigna radiata* (mung bean) seedlings. Plants were grown in pots under controlled environmental conditions and pre-treated with varying concentrations of *Bacillus amyloliquefaciens* D5-derived siderophores (5 μg/mL, 10 μg/mL, and 15 μg/mL) to assess dose-dependent effects on enzyme activity. Foliar treatment of siderophore solution at different concentrations was applied to each plant. Control treatments included untreated plants (no siderophore application) and pathogen-only treatments, where plants were inoculated with *Cercospora canescens* without prior siderophore treatment. Siderophore application occurred 24 h before pathogen inoculation. Enzyme activity was monitored from Day 0 to Day 8 post-inoculation. The inoculum was prepared by suspending *C. canescens* spores in a sterile solution, with a final concentration of 10^6^ spores/mL used for pathogen inoculation. The treatment groups were clearly defined as follows:

**Table:** 

Treatments	Description
C – Control	Untreated plant
CP – Control + Pathogen	Pathogen Inoculation
S5-Siderophore (5 μg)	Siderophore only (5 μg)
SP5 – Siderophore (5 μg) + Pathogen	Siderophore (5 μg) challenge inoculated with Pathogen
S10– Siderophore (10 μg)	Siderophore only (10 μg)
SP10- Siderophore (10 μg) + Pathogen	Siderophore (10 μg) challenge inoculated with Pathogen
Siderophore (15 μg)	Siderophore only (15 μg)
SP15- Siderophore (15 μg) + Pathogen	Siderophore (15 μg) challenge inoculated with Pathogen

Each treatment group consisted of 10 plants, and all experiments were conducted in triplicate to ensure statistical validity. This experimental design enabled the assessment of the effect of siderophore pre-treatment on plant defense enzyme activity in response to pathogen infection.

### Peroxidase activity

Peroxidase activity was measured as the oxidation of guaiacol using hydrogen peroxide as substrate at 470 nm as described by Hammerschmidt et al. (1982). One gram of plant tissue was homogenized in 2 mL of 0.1 M phosphate buffer (pH 7.0) at 4°C. The homogenate was centrifuged; the supernatant collected was used as enzyme source. The reaction mixture consisted of 0.5 mL of enzyme extract, 1.5 mL of 0.05 M pyrogallol, 1.5 mL of 1% H_2_O_2_ was incubated at room temperature. Changes in the absorbance at 470 nm were recorded at 30s interval for 3 min. Activity was expressed as the increase in absorbance at 470 nm/min/g of fresh tissue.

### Polyphenol oxidase activity

Polyphenol oxidase (PPO) activity was determined by Am (1954) using catechol as substrate. Formation of yellow colored product benzoquinone by the oxidation of catechol as substrate was measured at 495 nm. Reaction mixture consisted of 0.5 mL of enzyme extract, 1.5 mL of 0.1 M Sodium phosphate buffer (pH 6.5); to start the reaction 200 μL of 0.01 M catechol was added. Enzyme activity was expressed as units/ml.

### Chitinase activity

The modified method of Shimahara and Takiguchi (1988) was followed to prepare colloidal chitin. 10 g of chitin flakes from shrimp cells was mixed with 200 mL of concentrated HCL with continuous stirring at chilled temperature overnight. The mixture was then filtered through muslin cloth and dropped into 600 mL of chilled ethanol with rapid stirring on ice. The colloidal chitin was then collected and centrifuged at 8000 g for 30 min at 4°C. The pellet was then washed with distilled water till the pH was neutral. The filtrate was again filtered with whatman filter paper 1.0 and washed until the washing solution was neutral. The colloidal chitin was then stored at 4°C (Joe and Sarojini, 2017; Urja Pandya et al., 2014). Homogenize plant tissues using 0.1 M sodium citrate buffer, pH 5.0, centrifuge the homogenate at 13,000 rpm for 20 min, and collect the supernatant. As per Boller and Mauch (1988), colorimetric chitinase assay was carried out using colloidal chitin as substrate. 0.4 mL of supernatant was mixed with 10 μL of 1 M sodium acetate buffer (pH 4.0) to that 10 mg of colloidal chitin was added incubate the reaction mixture at 37°C for 2 h. Centrifuge the mixture to terminate the reaction at 8000 rpm for 5 min. 0.5 mL of supernatant was taken in a fresh tube to that 50 μL of 1 M phosphate buffer; pH 7.1 was added and incubated with 2 mL of dimethyl amino benzaldehyde (DMAB) for 220 min at 37°C. The absorbance was measured at 585 nm; the enzyme activity was expressed as Units/ml.

### Phenylalanine ammonia lyase activity

One gram of plant tissue was homogenized with 3 mL of chilled 0.1 M sodium borate buffer (pH 7.0) containing 1.4 mM *β*- mercaptoethanol and 0.1 g of polyvinylpyrrolidone (PVPP). The enzyme extract was filtered and centrifuged at 13,000 rpm for 20 min. 0.4 mL of supernatant was incubated with 0.5 mL of 0.1 M borate buffer (pH 8.8) and add 0.5 mL of 12 mM L-phenylalanine for 30 min at 30°C. Phenylalanine ammonia lyase activity was measured based on trans-cinnamic acid formation at 290 nm using L phenylalanine as substrate. The enzyme activity was expressed as Units/mL (Dickerson et al., 1984).

### Total phenols

Total phenols were estimated according to Zieslin and Ben-Zaken (1993). One gram of homogenized tissues of plant seedlings were macerated in 80% of methanol and agitated for 15 min at 70°C. In 1 mL of methanolic extract, 5 mL of distilled water was added to that 250 μL of 1 N Folin-Ciocalteau’s phenol Reagent (FCR) was mixed and kept the reaction mixture at 25°C. Measurement of blue color developed was read at 650 nm.

### SDS PAGE

Leaf proteins were extracted using an extraction buffer containing 1% SDS, 0.1 M Tris-Cl (pH 6.8), 2 mM EDTA-Na2, 20 mM DTT, and 2 mM PMSF. The homogenate was transferred to tubes and centrifuged at 15,000 rpm for 5 min. The supernatant was precipitated using equal volumes of 20% TCA-acetone. Leaf tissue (1 g) was homogenized in 4 mL of 10% (w/v) TCA-acetone, kept on ice for 5 min, and centrifuged again at 15,000 g for 5 min. The supernatant was discarded, and the pellet was washed with acetone until colorless. The pellet was dried and dissolved in SDS buffer (0.5% SDS, 50 mM Tris-Cl pH 6.8, 20 mM DTT) (Niu et al., 2018). Protein content was determined using the Bradford method (Bradford, 1976). The protein was stored at −80°C until further use. Pellets were dissolved in a buffer containing 10% glycerol, 2.3% SDS, 5% 2-mercaptoethanol, 0.25% bromophenol blue, and 63 mM Tris–HCl (pH 6.8), and heated at 95°C for 10 min. Twenty micrograms of protein from different treatments were mixed with 5 μL of sample buffer in a microfuge tube and boiled for 10 min at 95°C. Samples containing equal amounts of protein were loaded into the wells of polyacrylamide gels (Bio-Rad System). Broad-range molecular weight markers (Bangalore Genei, India) were used, and electrophoresis was carried out at a constant voltage of 75 volts for 2 h. The gels were stained with 0.2% Coomassie Brilliant Blue (R250) solution.

### Native PAGE

The isoform profile of PO and PPO were examined by native PAGE (Laemmli, 1970). For all treatments, plants were pre-inoculated with siderophores 24 h before pathogen inoculation. Plant samples for biochemical enzyme assays (PO and PPO activity) were collected on Day 6 post-pathogen inoculation, which corresponds to the peak of enzyme activity. Plant samples were collected at the 6th day of pathogen challenge for PO and PPO, respectively, from 4 treatments C- Control, P – Pathogen, S- siderophore (50 μg) and siderophore (50 μg) + pathogen. The protein extract was prepared by homogenizing 1 g of plant sample in 2 mL of 0.1 M sodium phosphate buffer pH 7.0 and centrifuged at 13,000 g for 25 min at 4°C. After electrophoresis, PO isoforms were visualized by soaking the gels in staining solution containing 0.05% benzidine and 0.03% H_2_O_2_ in acetate buffer (20 mM, pH 4.2) (Nadolny and Sequeira, 1980). For assessing PPO isoforms profile, the gels were equilibrated for 30 min in 0.1% p-phenylenediamine followed by addition of 10 mM catechol in the same buffer (Jayaraman et al., 1987).

### Pre-treatment of plants for molecular analysis

Foliar treatment of 50 µg/mL of purified siderophore was given to the mung bean plants prior to *Cercospora canescens* inoculation. The fungal inoculation was done at concentration of 10^6^ spores/ml for inoculation. Four treatments as *C. canescens* only, Siderophore only, Siderophore with *C. canescens* and Control (untreated) were maintained. Plants (3 plants from each replication) were uprooted at 6 days post-inoculation (dpi) and used for further PCR analysis.

### Reverse transcription PCR

Total RNA was isolated from the treatments manually by Trizol C method. The cDNA synthesis was carried out using the Mastercycler X40 thermo cycler by Eppendorf and Quantbio qscript cDNA synthesis kit which included nuclease free water, qScript reaction mixture and qScript RT (Reverse transcriptase). 5 μL of RNA sample was added to the mixture at concentration of 1 μg and the total reaction mixture was of 20 μL. The primers for various defense-related genes were designed using the sequences of mung bean defense genes available in the NCBI database (Dubey et al., 2018). The gene-specific primers (Table 1) were used for quantitative PCR analysis. The actin gene was used as the housekeeping gene for the normalization of the expression data (Dubey et al., 2018; Azeem et al., 2021; Nivya and Shah, 2024).

**Table 1:** List of primers for PCR analysis.

Genes	Accession number	Primers	Amplicon size
PR-10	AY792956.1	F-GACGAGGCAAACTTGGGATA	217
		R-CAGCCTTGAAAAGTGCATCA	
Epoxide hydrolase	HQ316148.1	F-AACTGGGGGCCTCAACTACT	243
		R-TCCTCTGCAGCTTCTTGGTT	
Catalase	D13557.1	F-AGTTCCCCATACCTCCTGCT	219
		R-GAGAACGGTCAGCCTGAGAC	
Calmodulin	DQ778070.1	F-AACAAGGAGGTCGTGGTGTC	300
		R-ATGCCGATCACAAAACAACA	
Actin	AF143208.1	F-TCGTGTGGCTCCTGAAGAAC	230
		R-AGATTGCATGTGGAAGGGCA	

### Quantitative real time PCR

The real-time PCR was conducted using the StepOnePlus Real-Time PCR System (Applied Biosystems). Each reaction included 10 μL of RealQ Flex 2X Master Mix, 0.4 μL each of forward and reverse primers (Primer A and Primer B), 0.3 μL of ROX dye, and 7.9 μL of PCR grade water, with 1 μL of cDNA template added to initiate the PCR process. The ROX dye was diluted from a stock concentration of 200 μM to 300 nM by a 1:10 dilution in PCR grade water, resulting in a final volume of 5 μL per 50 μL of ROX dilution. The PCR conditions for PR10, epoxide hydrolase, calmodulin and catalase genes were: initial denaturation at 95°C for 3 min, followed by 45 cycles of denaturation at 95°C for 10 s, annealing at 60°C for 20 s, extension at 62°C for 20 s, and plate reading at 62°C. For the calmodulin gene, the conditions were: initial denaturation at 95°C for 4 min, followed by 45 cycles of denaturation at 95°C for 10 s, annealing at 60°C for 30 s, and plate reading at 62°C. Each treatment sample was run in triplicate. The relative expression ratios of the defense genes were normalized using actin as a reference gene. The crossing point (C(T)) values of each sample were compared to a control sample (mock-inoculated) to determine relative expression (Patil et al., 2021; El-Maraghy et al., 2020).

### Statistical analysis

For each of the investigated biochemical parameters from control and treated samples, three separate replication sets were conducted. All the experimental measurement values were expressed as means of three measurements ± standard error. The significance of the differences between the mean values of control and treatments were evaluated using ANOVA and *post hoc* test Dunnett’s Multiple Range Test and Tukey’s test using GraphPad Prism 5 software.

## Results and discussion

### Siderophore induced biochemical enzyme activity

The effects of siderophore treatments on biochemical enzyme activities and their role in plant defense against *C. canescens* were assessed.

Total phenols increased in all treatments except the control (untreated), with the highest values observed in the S15 and SP15 treatments (80 and 100 μg/mL, respectively) on Day 6. The SP15 treatment exhibited a synergistic effect of siderophore and pathogen, leading to significantly enhanced phenol production, likely due to a combination of pathogen-induced and siderophore-mimicked immune responses. The peroxidase activity was highest in SP15 (0.563 units on Day 6), reflecting an induced defense response against *C. canescens* invasion. Siderophore treatments, particularly at higher concentrations (S10, S15), resulted in significant increases in peroxidase activity, while the control remained unchanged, as peroxidase is constitutively active under normal conditions.

Polyphenol oxidase (PPO) activity was highest in SP15 and S10 (0.1 and 0.08 U/mL, respectively), with peak levels observed on Day 6 and Day 8. This increase suggests that PPO plays a key role in the plant’s defense mechanism against stressors like *C. canescens*, with siderophores enhancing the availability of substrates for PPO activity. Phenylalanine ammonia lyase (PAL) activity peaked in the SP15 group (100 U/mL) on Day 2, and increased progressively in the S10 and S15 treatments. PAL activity, which is vital for synthesizing antimicrobial compounds, was upregulated in response to both the siderophore and pathogen, indicating an enhanced defense response.

**Figure 1:**
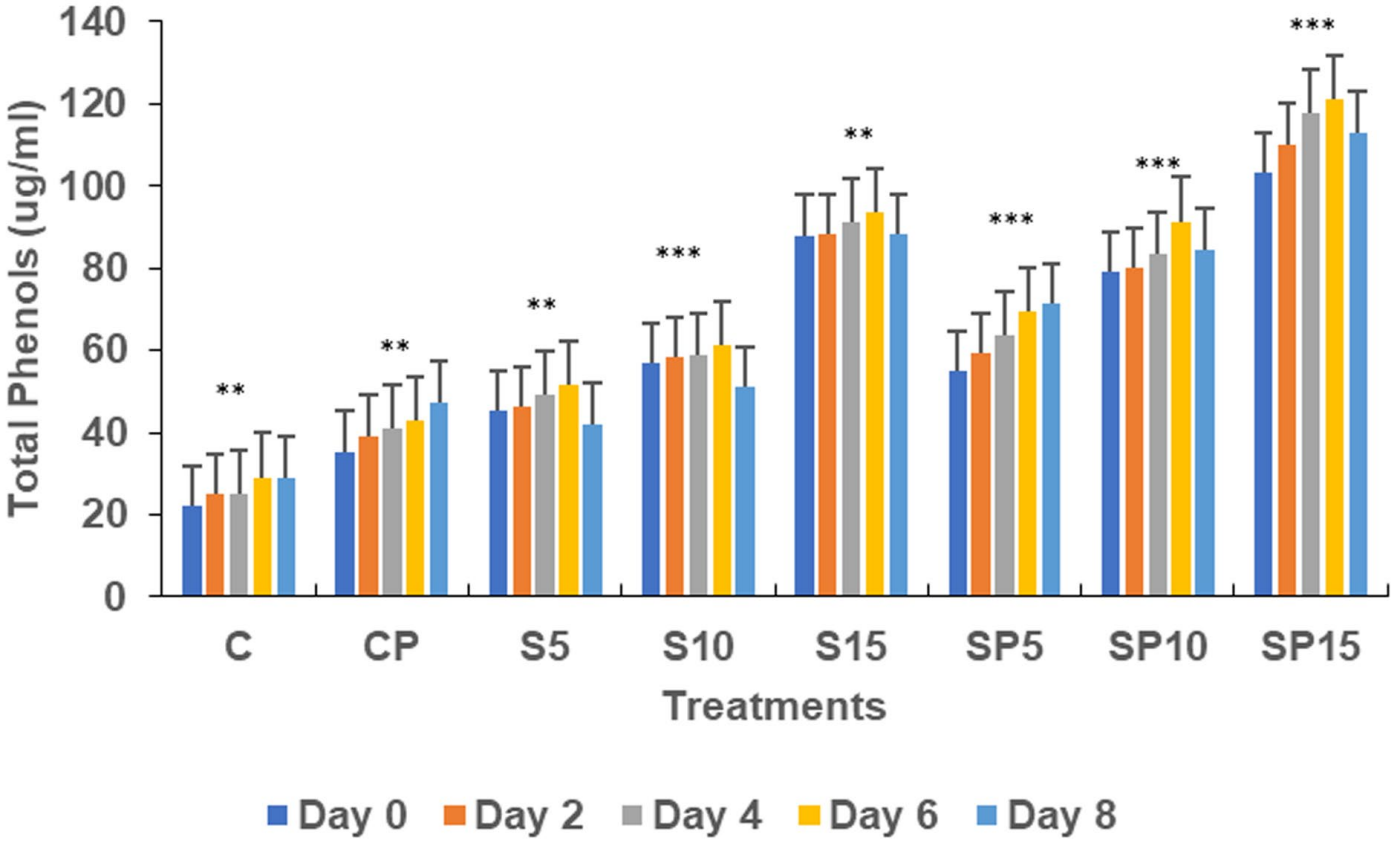
Total phenols under various treatments. Statistical analysis was determined using ANOVA followed by Dunnett’s Multiple Range Test. C, Control; S, Siderophore (5, 10, 15 μg); SP, Siderophore + Pathogen (5, 10, 15 μg). The asterisks indicate significant differences relative to the Control group (C): ns = *p* > 0.05, *p* ≤ 0.05, *p* ≤ 0.01, *p ≤ 0.001.

**Figure 2:**
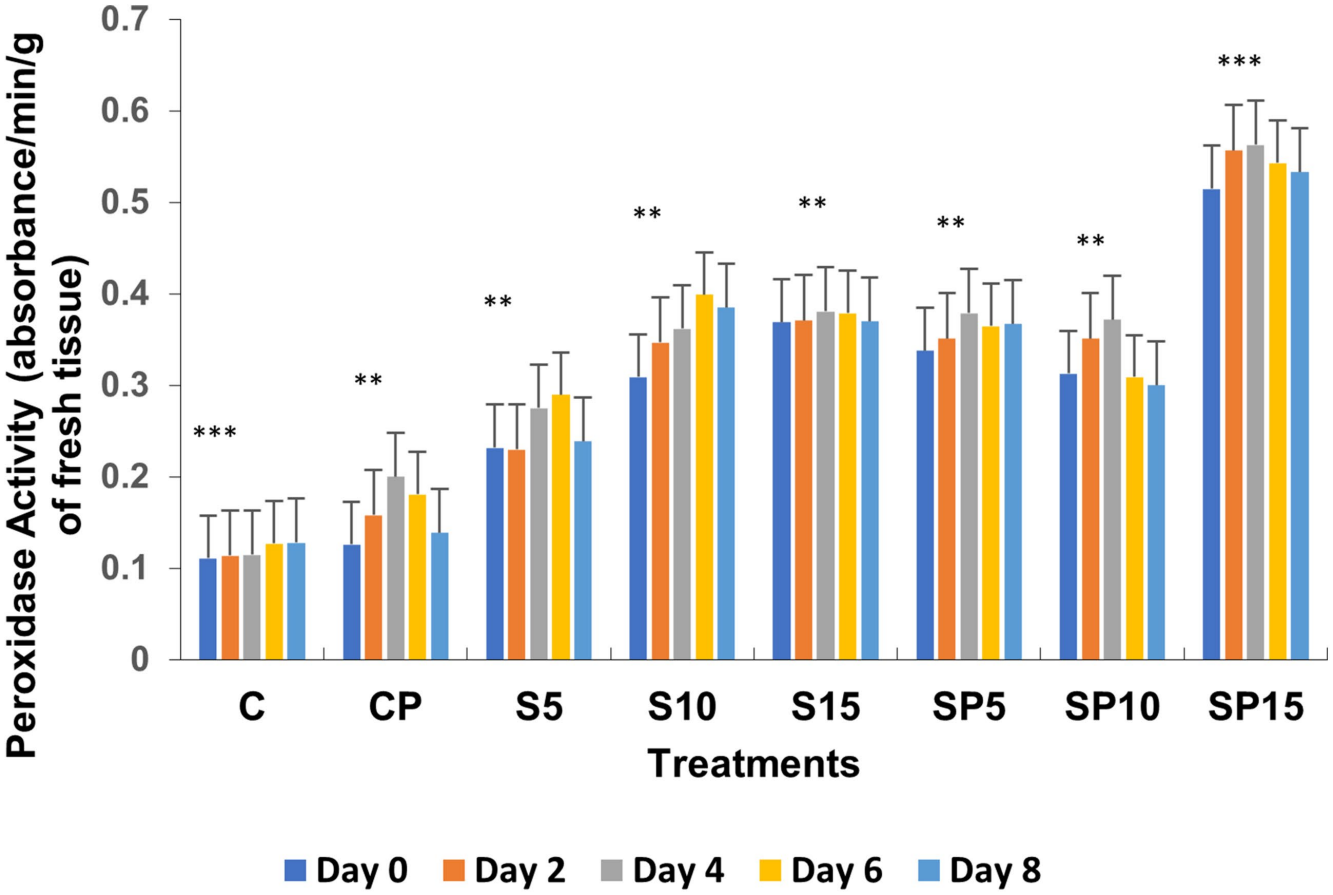
Peroxidase activity under various treatments: C, Control; S, Siderophore (5, 10, 15 μg); SP, Siderophore + Pathogen (5, 10, 15 μg). Statistical significance was determined using ANOVA followed by Dunnett’s Multiple Range Test. The asterisks represent comparisons relative to the Control group (C): ns = *p* > 0.05, *p* ≤ 0.05, *p* ≤ 0.01, *p ≤ 0.001.

**Figure 3:**
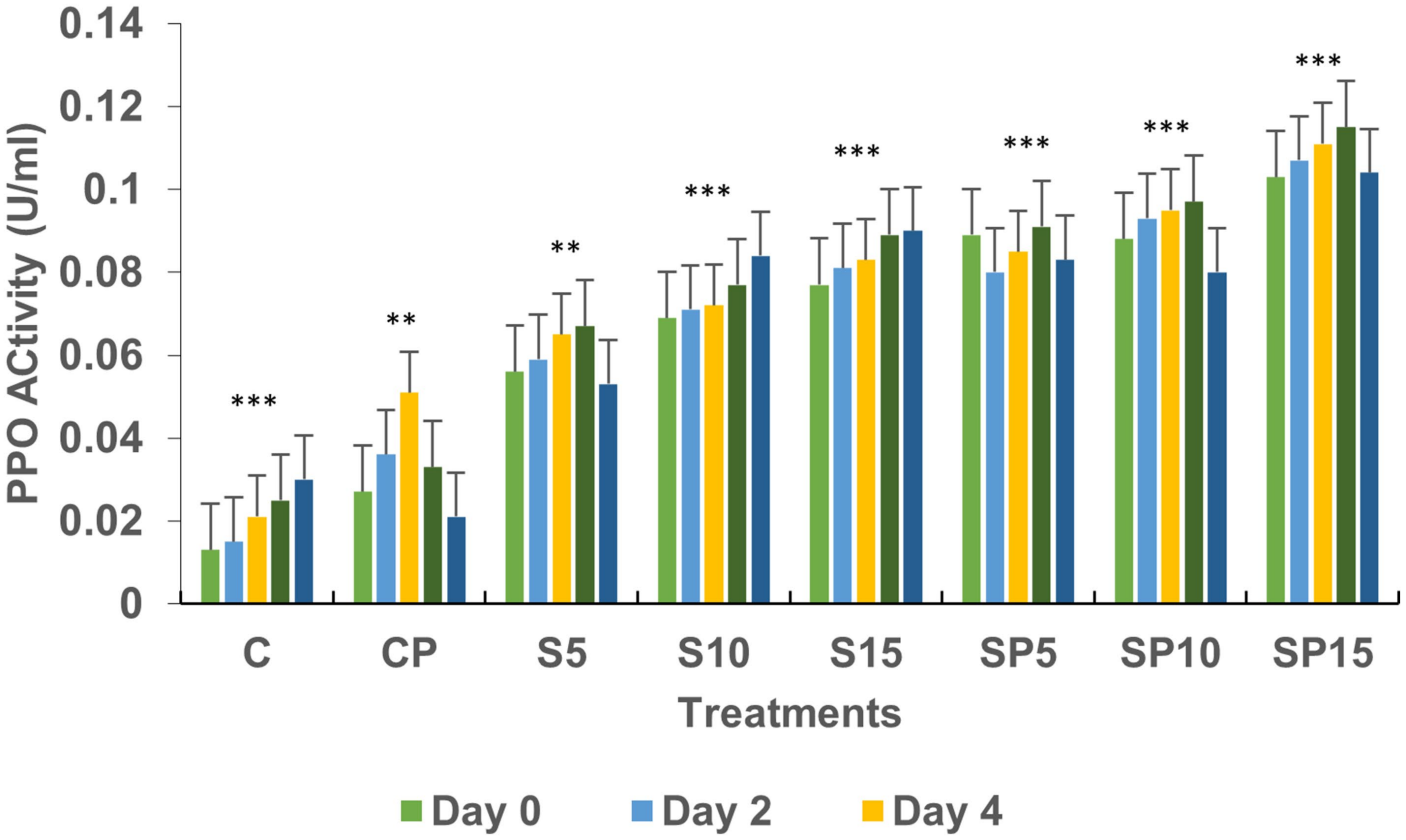
Polyphenol oxidase activity under various treatments: C, Control; S, Siderophore (5, 10, 15 μg); SP, Siderophore + Pathogen (5, 10, 15 μg). Statistical significance was determined using ANOVA followed by Dunnett’s Multiple Range Test. Asterisks represent comparisons relative to the Control group (C): *ns* = *p* > 0.05, *p* ≤ 0.05, *p* ≤ 0.01, *p ≤ 0.001.

**Figure 4:**
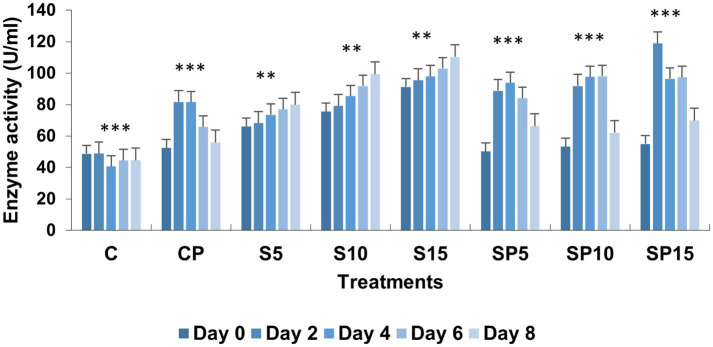
Phenylalanine ammonia lyase activity under various treatments: C, Control; S, Siderophore (5, 10, 15 μg); SP, Siderophore + Pathogen (5, 10, 15 μg). Statistical significance was determined using ANOVA followed by Dunnett’s Multiple Range Test. Asterisks indicate comparisons relative to the Control group (C): ns = *p* > 0.05, *p* ≤ 0.05, *p* ≤ 0.01, *p ≤ 0.001.

**Figure 5:**
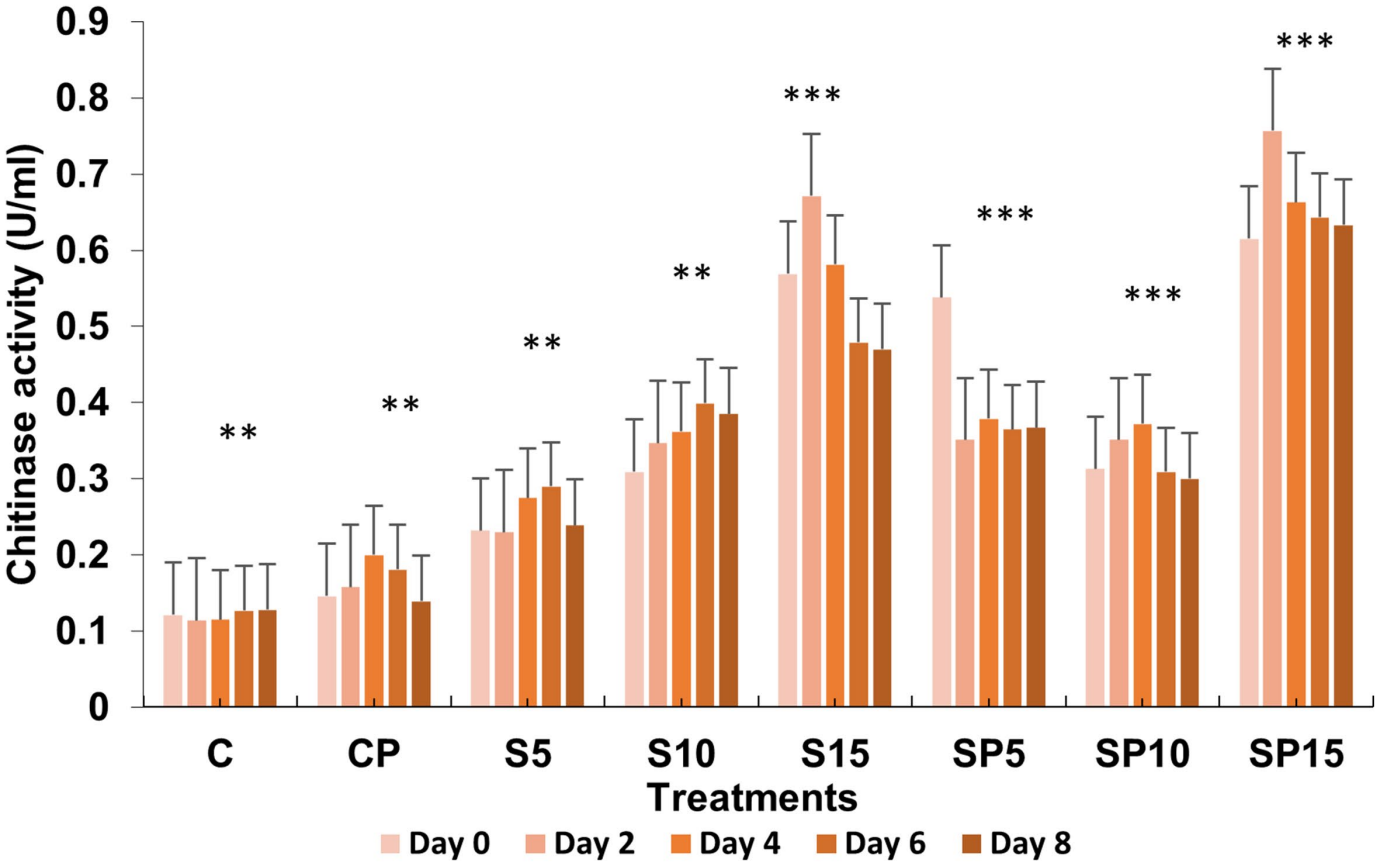
Chitinase activity under various treatments: C, Control; S, Siderophore (5, 10, 15 μg); SP, Siderophore + Pathogen (5, 10, 15 μg). Statistical significance was determined using ANOVA followed by Dunnett’s Multiple Range Test. Asterisks denote comparisons relative to the Control group (C): ns = *p* > 0.05, *p* ≤ 0.05, *p* ≤ 0.01, *p ≤ 0.001.

Chitinase activity was highest in the combined treatment (Siderophore + *C. canescens*) on Day 4, with sustained elevation observed through Day 8. This suggests a synergistic interaction between siderophores and the pathogen, enhancing chitinase-mediated defense mechanisms and also indicate a potential crosstalk of defense pathways activated by both siderophore and pathogens. Overall, these results demonstrate that siderophores, both individually and in combination with pathogens, can induce significant defense-related biochemical changes in plants, with higher concentrations showing more pronounced effects. This study supports the potential of siderophore applications for enhancing plant resistance to pathogens. Similar findings were reported by Ashajyothi et al. (2023), where purified hydroxamate siderophores from *Pseudomonas putida* B25 induced increased peroxidase, PPO, and phenol levels in response to *Magnaporthe oryzae* infection in rice (Figures 1–5).

### SDS PAGE for protein expression analysis

**Figure 6:**
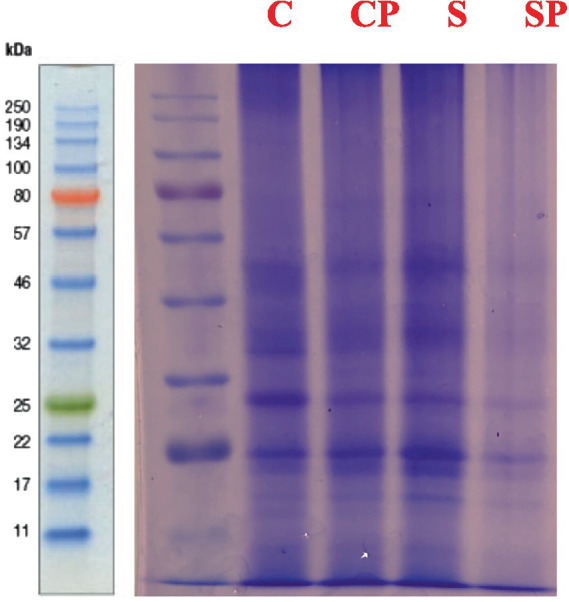
SDS PAGE analysis of proteins under various treatments; C, Control; P, Pathogen only; S, siderophore only; SP, siderophore + pathogen.

The protein banding patterns was studied in various treatments treated with only siderophores and in presence of pathogen along with positive control (untreated plant) and negative control (pathogen only). The result revealed the presence of 80 KDa; 32-46KDa; 25KDa, 22KDa bands in Control, 80KDa, 46-50KDa; 32-46KDa; 25KDa, 22KDa, 17KDa bands in Pathogen treatment, Siderophore – 46-50KDa; 32-46KDa; 25KDa, 22KDa, 17KDa and Siderophore + Pathogen– 46-50KDa; 32-46KDa; 25KDa, 22KDa, 17KDa. Additional bands were observed in pathogen treated plants at 45-50Kda and 17Kda (Figure 6). Similar banding patterns to pathogen were observed in siderophore treated plants indicating some proteins may be broadly induced in response to different elicitors as part of a general stress response mechanism in plants. These stress-responsive proteins may be commonly detected in samples treated with siderophores or pathogens, contributing to the observed similarity in protein bands. The response to siderophore treatment may involve cross-talk between defense mechanisms triggered by pathogen recognition and nutrient signaling pathways activated in response to siderophores (Devi et al., 2024a, b). This cross-talk could lead to convergence in protein expression patterns observed in siderophore-treated and pathogen-treated sample. Similar banding pattern was observed in siderophore + pathogen treatment indicating that siderophores and pathogens have synergistic effects on plant responses, leading to similar changes in protein expression or modification. The combination of siderophores and pathogens may potentiate certain defense mechanisms, resulting in overlapping banding patterns compared to individual treatments. Siderophores and pathogens may activate overlapping signaling pathways in plants. These pathways could converge on shared downstream targets, leading to similar changes in protein expression or modification in response to siderophore + pathogen treatment as observed in individual treatments. The SDS-PAGE analysis of protein in biocontrol agents treated plant showed eight proteins with molecular weight of 14, 23, 30, 35, 45, 50, 60, and 98 kDa. In healthy plants only six proteins (excepting 14 and 98 kDa) appeared in biocontrol agents treated plant two additional new proteins appeared (Ganeshamoorthi et al., 2008).

### Native PAGE

**Figure 7:**
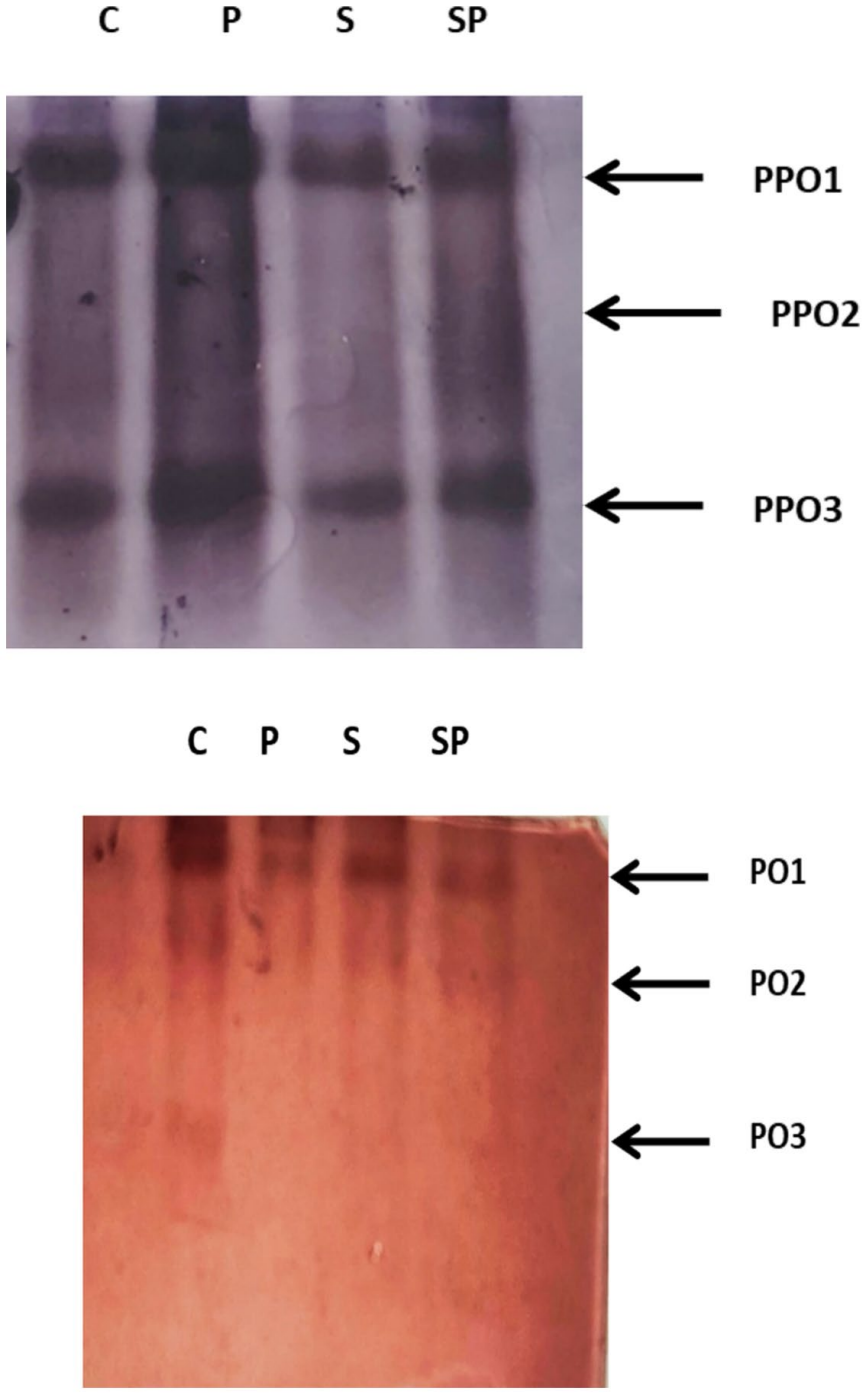
PO (Peroxidase) and PPO (Polyphenol Oxidase) isoform staining. C, Control; P, Pathogen only; S, siderophore only; SP, siderophore + pathogen.

The Peroxidase (PO) and Polyphenol Oxidase (PPO) isoforms were studied for the treatments Control, Pathogen, Siderophore (50µg) and Siderophore + Pathogen. Similar isoform banding was observed in the treatments that is PPO isoform 1, PPO isoform 2 and PPO isoform 3. Some isoforms of PPO may be constitutively expressed in plants where they are present under normal, untreated conditions (control). These isoforms may play roles in various physiological processes, such as defense against herbivores or pathogens, or in the oxidation of phenolic compounds involved in plant development. Pathogen infection, siderophore treatment, or their combination may induce changes in PPO expression levels or activity as part of the plant defense responses. However, these treatments may not necessarily result in the expression of entirely new isoforms of PPO. Instead, they may modulate the expression levels or post-translational modifications of existing isoforms. PO isoforms 1, 2 were expressed in all treatments but PO isoform 3 was only found in control treatment as each peroxidase isoform may have a distinct role in responding to different types of stress. The treatment applied may only activate two of the isoforms observed in the control because they are more directly involved in responding to the specific stressor introduced by the treatment, while the third isoform may be less relevant to the treatment-induced stress response (Figure 7). The native gel electrophoresis of enzyme extracts from biocontrol agent-treated plants revealed that the combination treatments of biocontrol agents led to higher induction of peroxidase (PO) and polyphenol oxidase (PPO) isoforms. Specifically, the combination of *Pf1* + *Py15* + *Bs16*, *Pf1* + *Bs16*, and *Pf1* + *Py15* resulted in stronger induction of both PO and PPO isoforms. In contrast, the inoculated control treatment showed lesser induction of PO isoforms, while the healthy and inoculated control treatments exhibited mild induction of PPO isoforms with lower intensity. This suggests that the combination of the three biocontrol agents is more effective in enhancing enzyme activity and isoform expression in mulberry plants (Ganeshamoorthi et al., 2008).

### Quantitative PCR

Catalase-mediated detoxification of ROS helps plants to limit the spread of pathogens by preventing oxidative damage to host cells. Additionally, the production of ROS by the plant in response to pathogen recognition can act as a signaling molecule to activate defense pathways and reinforce the plant immune response (Dubey et al., 2018). In the absence of any treatment or stressor, the *CAT* gene expression remains at baseline levels. This indicates that under normal conditions, the plant maintains a steady-state level of *CAT* expression necessary for basic cellular functions. The upregulation of *CAT* gene expression by approximately 4.4-fold in response to pathogen treatment suggests a significant induction of the antioxidant defense system. Pathogen infection often triggers the production of ROS as part of the plant defense response, leading to oxidative stress. The substantial upregulation of catalase gene expression by approximately 21.1-fold in response to siderophore treatment indicates a strong activation of the antioxidant defense mechanism. Siderophores are iron-chelating compounds that can induce oxidative stress in plants due to their ability to generate ROS as byproducts. The significant increase in *CAT* expression suggests that plants respond robustly to siderophore-induced oxidative stress by enhancing ROS detoxification capacity through catalase-mediated pathways. The synergistic effect observed in the combined treatment of siderophore and pathogen, resulting in a further upregulation of catalase gene expression by approximately 25.9-fold, suggests an amplified activation of catalase expression. Lipid peroxides are generated as by-products of oxidative stress, particularly under conditions of pathogen infection, drought, or high light intensity. Epoxide hydrolases participate in the metabolism of lipid peroxides, facilitating their conversion into less reactive and cytotoxic compounds. This process helps to alleviate oxidative damage and maintain cellular homeostasis under stress conditions (Tripathi et al., 2023).

The expression of the *EH* gene is highly overexpressed in response to pathogen treatment as part of the plant defense response to pathogen infection. The expression of the *EH* gene is moderately upregulated approximately 77.3 times in response to siderophore treatment compared to the control condition while not as pronounced as the response to pathogen treatment, this increase indicates a significant induction of gene expression in response to siderophore application, possibly as a protective and adaptive response to siderophore-induced stress.

The combined treatment of siderophore and pathogen leads to a very high expression or upregulation of *EH* gene as compared to control condition. This synergistic effect suggests an intensified response to the combined stressors resulting in a more pronounced induction of gene expression compared to either treatment alone. Following an inducing treatment, plant resistance may manifest in three ways: (i) defenses are activated immediately with no further change after pathogen challenge, (ii) initial defenses are amplified or diversified upon pathogen challenge, or (iii) defenses remain dormant until the pathogen triggers their activation (Walters et al., 2005).

Calcium/calmodulin-dependent protein kinases (CCaMKs) and other calmodulin-binding proteins phosphorylate and activate transcription factors such as WRKY and MYB, which regulate the expression of defense-related genes (Yuan et al., 2022). Calmodulin-dependent protein kinases (CDPKs) directly phosphorylate and activate enzymes involved in defense responses, such as pathogenesis-related (PR) proteins, defense-related enzymes, and antimicrobial compound. Calmodulin participates in the regulation of cell wall modifications and modulates the activity of enzymes involved in the synthesis and remodeling of cell wall components, such as callose synthases, pectin methylesterases, and xyloglucan endotransglucosylases/hydrolases (XTHs), which contribute to the strengthening of cell walls and physical barriers against invading pathogens (Wang et al., 2021). The *CAL* gene was moderately regulated in pathogen treatment as compared to other treatments. The higher gene expression was observed in siderophore pathogen treatment followed by siderophore treatment. The higher gene expression in siderophore + pathogen again attributes to the synergistic effect of both elicitors. This creates an amplified response to the presence of combined elicitors. The siderophore has the ability to induce upregulation of *CAL* gene owing to its adaptive and protective response toward pathogen attack.

**Figure 8:**
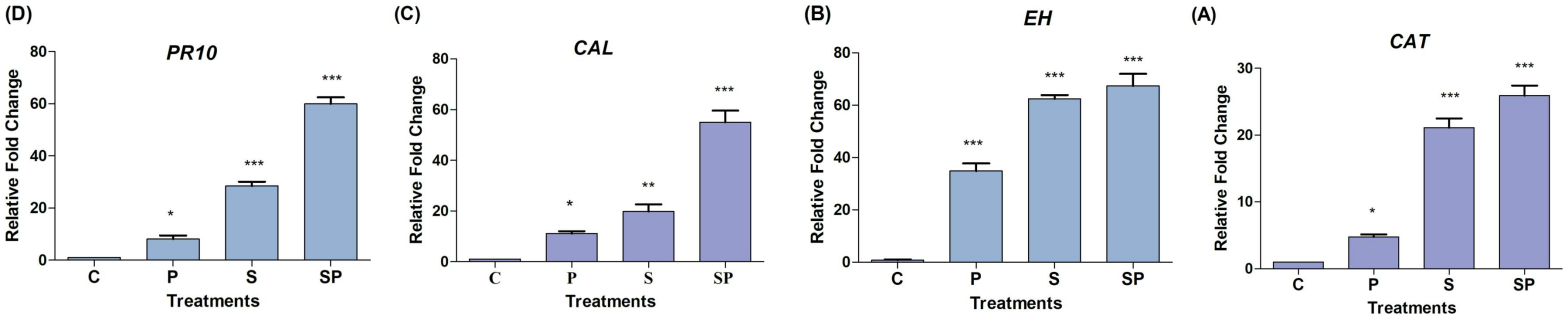
Relative fold change under various treatments: Control (C), Siderophore (S), Pathogen (P), and Siderophore + Pathogen (SP). Statistical analysis was performed using one-way ANOVA followed by Tukey’s Test to compare treatment groups against the control. ns indicates no significant difference (*p* > 0.05), *p ≤ 0.05, **p ≤ 0.01, ***p ≤ 0.0.

*PR-10* proteins are a subset of the pathogenesis-related (PR) protein family, which comprises several groups of proteins that are associated with plant defense mechanisms against pathogens (Dos Santos and Franco, 2023). The *PR10* gene was upregulated in both siderophore and siderophore + pathogen treatments owing to a more heightened response to pathogen invasion and enhanced protection to the plant. The pathogen treatment shows moderate levels of PR10 expression as plants may prioritize responses to different elicitors based on the perceived severity and nature of the stress. While pathogen infection is a potent trigger of defense responses, siderophore treatment or combined stress may elicit additional or stress signals that further enhance gene expression. Gene expression patterns can vary over time, with different genes being induced or repressed at different stages of the plant’s response to stress. The effects of chemical elicitors salicylic acid (SA) and jasmonic acid (JA) on expression of defense genes PR 10, epoxide hydrolase (EH), catalase and calmodulin with infection by *Rhizoctonia solani* were analyzed using qPCR at 1–4 days post inoculation/application (dpi) in highly susceptible (HS; Ratna) and moderately resistant (MR; HUM1) varieties of mungbean suggesting the upregulation of these genes in both the varieties (Tripathi et al., 2023; Figure 8).

## Conclusion

In conclusion, the results of this study confirm that siderophores produced by *Bacillus amyloliquefaciens* enhance plant resistance through the activation of key defense mechanisms. Our findings demonstrate that siderophore treatments significantly increase peroxidase (POD), polyphenol oxidase (PPO), phenylalanine ammonia-lyase (PAL), and chitinase activities, indicating a strong induction of plant immune responses. These enzyme activities, along with changes in gene expression, such as upregulation of catalase and epoxide hydrolase, suggest that siderophores activate antioxidant defense systems and alleviate oxidative stress, further supporting their role in boosting plant immunity against pathogens. Notably, the synergistic effects observed in plants treated with both siderophores and pathogens highlight the potential of siderophores to amplify plant defense responses, making them a promising tool for crop protection.

This study suggests that siderophore-producing bacteria could serve as an eco-friendly alternative to chemical fungicides and also that siderophores alone act as elicitor of defense responses and are potential biostimulants, reducing their use in agriculture while improving crop resilience. However, limitations such as the controlled experimental setting and the need for field validation must be addressed before broad application. Future research should focus on conducting field trials to assess the effectiveness of siderophore-based treatments under natural conditions and explore their broader applications across various crops and environmental scenarios. Additionally, optimizing siderophore production, improving downstream processing, and exploring genetic engineering techniques could facilitate the commercialization of these bio-based solutions for sustainable agriculture.

## Data Availability

The original contributions presented in the study are publicly available. This data can be found here: https://www.ncbi.nlm.nih.gov, accession number OQ536061.
