# Supplementary figures and images for: Fortifying plant fortresses: siderophores in defense against Cercospora leaf spot disease in Vigna radiata L
Source: Front Microbiol. 2025 Jan 27;15:1492139. doi: 10.3389/fmicb.2024.1492139 (PMC11808597; doi:10.3389/fmicb.2024.1492139)

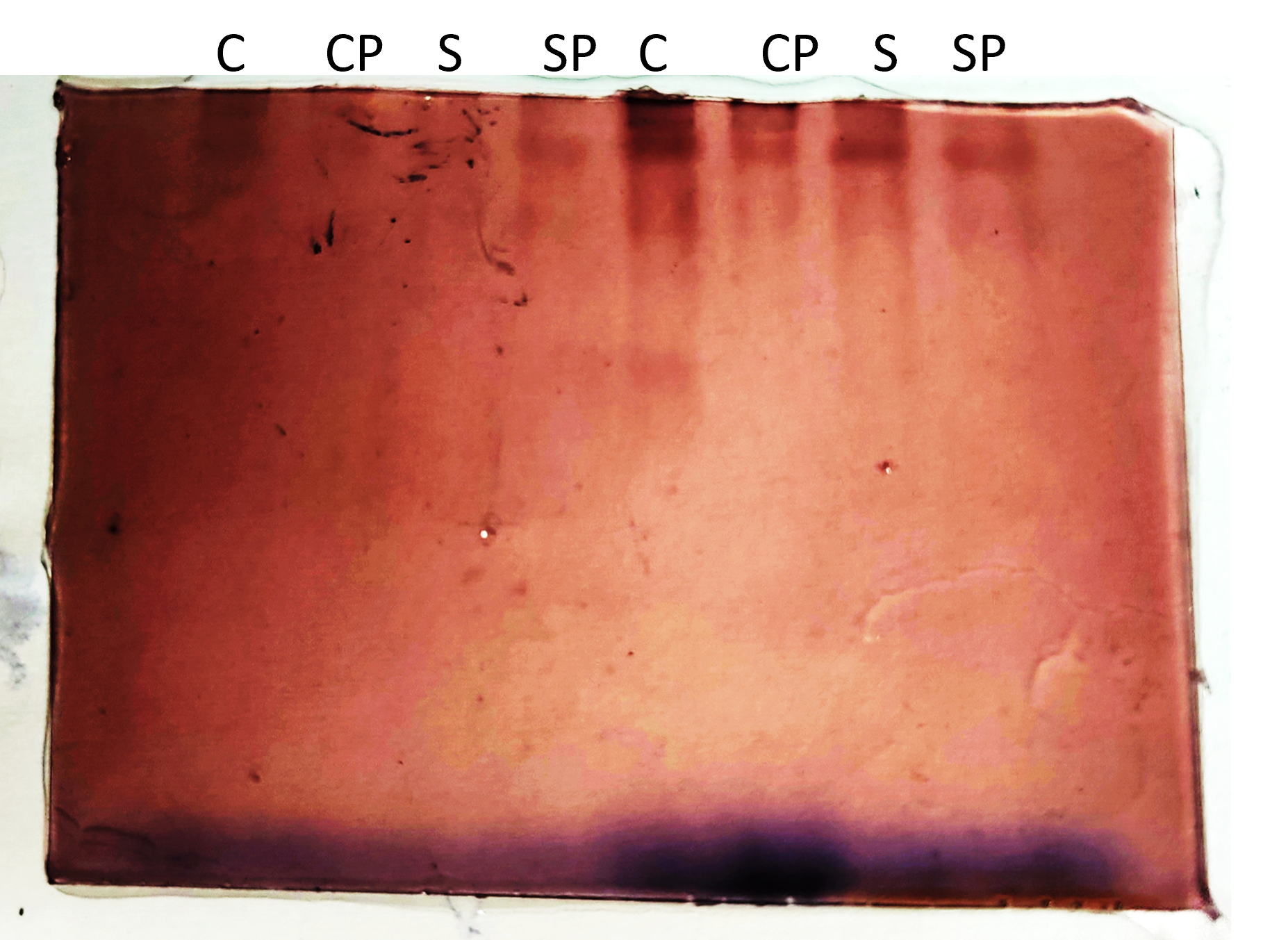

Supplement: Supplementary file 1 [file Image_1.tif]

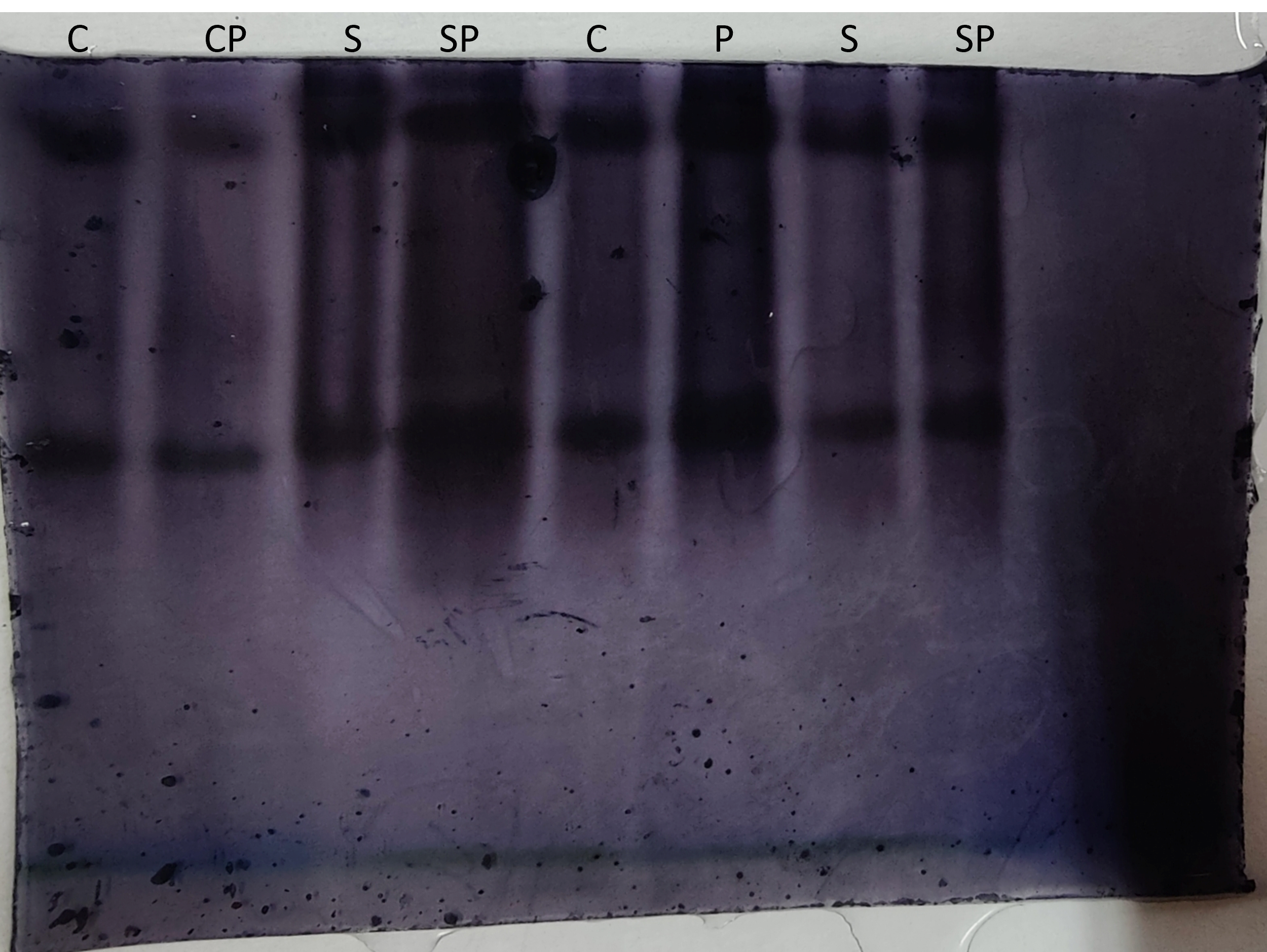

Supplement: Supplementary file 2 [file Image_2.tif]

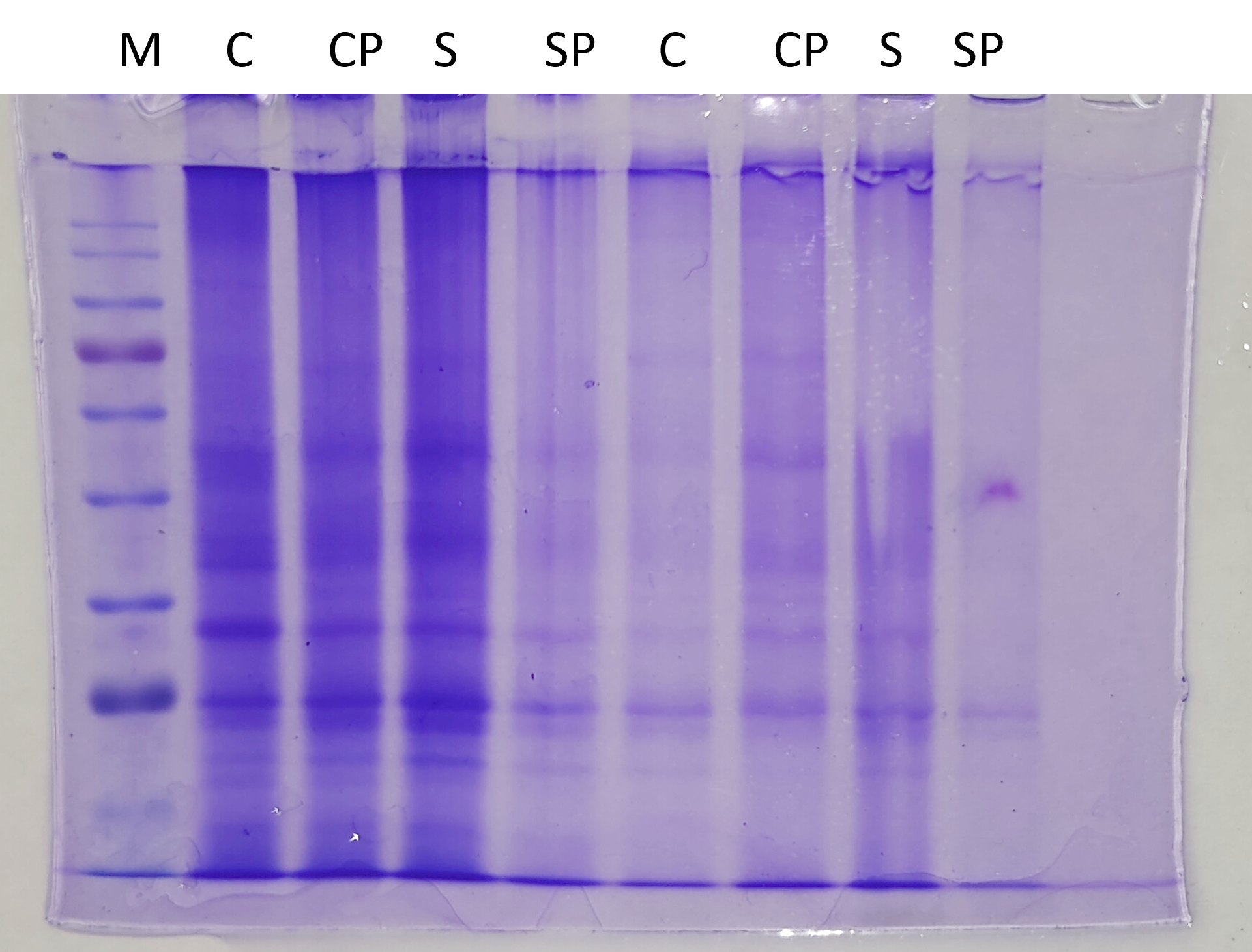

Supplement: Supplementary file 3 [file Image_3.tif]
